# Paediatric and Neonatal Pain—Editorial

**DOI:** 10.1002/pne2.12004

**Published:** 2019-05-22

**Authors:** Elaine M. Boyle

We are delighted to announce the launch of *Paediatric and Neonatal Pain*, a double‐blind peer‐reviewed multidisciplinary journal focusing on cutting‐edge research into paediatric and neonatal pain management, clinical practice and analgesia. This exciting new journal will provide scientists, researchers and clinicians with a platform to raise the profile of a fundamental and multifaceted part of neonatal and paediatric practice.

The approach to and management of pain in the paediatric population is very different from that in adults and demands a different set of skills and therapies. The immature anatomy and physiology of the neonate and child mean that different cautions must be exercised when considering physical and pharmacological treatments. Both pain and its management can have profound effects on children's long‐term health and development that are not relevant for older people. With increasing acknowledgement of these factors, there has emerged a growing need for a focused and specialised journal, designed to disseminate information and highlight important issues that are specific to pain in babies, children and young people, as well as their parents, families and carers, including healthcare professionals. *Paediatric and Neonatal Pain* will provide this much‐needed opportunity to discuss and inform on important clinical and scientific issues and highlight work that is being carried out around the world in this area.

Painful procedures in the neonatal period, infancy and childhood are undesirable, but often unavoidable, and painful conditions can occur at any age. Understanding mechanisms of pain in babies, children and young people, and how it differs from our knowledge of mechanisms in adults, is important to allow the development of new therapies and drugs that are suitable for use in the young. Assessment and management of pain in these vulnerable populations is well‐recognised to be a challenge. For the pre‐verbal infant, accurate assessment of pain is key to appropriate management. For young children, developing ways for them to convey the pain they are experiencing is crucial. Managing pain effectively and safely, whilst minimising the short‐ and long‐term adverse effects of both the pain itself and the measures we use to prevent and treat it, is paramount.

Recent years have seen increasing interest in and investigation of such issues in babies and children, but there is much still to learn and to be discovered. There are many aspects that demand further exploration, including not only the scientific aspects of pain, its physical effects, and strategies for management, but also the psychological impact of the experience of pain, both for the individuals themselves and also for those caring for them. *Paediatric and Neonatal Pain* is well placed to allow authors to summarise the state of the art in reviews and to showcase the current and future work that will ultimately guide patient management.

Our aim for *Paediatric and Neonatal Pain* is to span medicine and nursing and allied healthcare professions, serving healthcare workers and the paediatric care research community. *Paediatric and Neonatal Pain* welcomes submission of papers focussed on any aspect of pain in infants or children. We encourage clinicians and scientists from any discipline involved in the investigation or management of pain in infants and children to send their papers to be considered for publication.

